# Examining Internet and eHealth Practices and Preferences: Survey Study of Australian Older Adults With Subjective Memory Complaints, Mild Cognitive Impairment, or Dementia

**DOI:** 10.2196/jmir.7981

**Published:** 2017-10-25

**Authors:** Haley M LaMonica, Amelia English, Ian B Hickie, Jerome Ip, Catriona Ireland, Stacey West, Tim Shaw, Loren Mowszowski, Nick Glozier, Shantel Duffy, Alice A Gibson, Sharon L Naismith

**Affiliations:** ^1^ Brain and Mind Centre University of Sydney Camperdown Australia; ^2^ Charles Perkins Centre School of Psychology University of Sydney Camperdown Australia; ^3^ Brain and Mind Centre Faculty of Medicine University of Sydney Camperdown Australia; ^4^ Charles Perkins Centre Faculty of Health Science University of Sydney Camperdown Australia; ^5^ Charles Perkins Centre Faculty of Medicine University of Sydney Camperdown Australia; ^6^ Boden Institute of Obesity, Nutrition, Exercise & Eating Disorders University of Sydney Camperdown Australia

**Keywords:** eHealth, dementia, mild cognitive impairment, Internet, Alzheimer disease

## Abstract

**Background:**

Interest in electronic health (eHealth) technologies to screen for and treat a variety of medical and mental health problems is growing exponentially. However, no studies to date have investigated the feasibility of using such e-tools for older adults with mild cognitive impairment (MCI) or dementia.

**Objective:**

The objective of this study was to describe patterns of Internet use, as well as interest in and preferences for eHealth technologies among older adults with varying degrees of cognitive impairment.

**Methods:**

A total of 221 participants (mean age=67.6 years) attending the Healthy Brain Ageing Clinic at the University of Sydney, a specialist mood and memory clinic for adults ≥50 years of age, underwent comprehensive clinical and neuropsychological assessment and completed a 20-item self-report survey investigating current technology use and interest in eHealth technologies. Descriptive statistics and Fisher exact tests were used to characterize the findings, including variability in the results based on demographic and diagnostic factors, with diagnoses including subjective cognitive impairment (SCI), MCI, and dementia.

**Results:**

The sample comprised 27.6% (61/221) SCI, 62.0% (137/221) MCI, and 10.4% (23/221) dementia (mean Mini-Mental State Examination=28.2). The majority of participants reported using mobile phones (201/220, 91.4%) and computers (167/194, 86.1%) routinely, with most respondents having access to the Internet at home (204/220, 92.6%). Variability was evident in the use of computers, mobile phones, and health-related websites in relation to sociodemographic factors, with younger, employed respondents with higher levels of education being more likely to utilize these technologies. Whereas most respondents used email (196/217, 90.3%), the use of social media websites was relatively uncommon. The eHealth intervention of most interest to the broader sample was memory strategy training, with 82.7% (172/208) of participants reporting they would utilize this form of intervention. Preferences for other eHealth interventions varied in relation to educational level, with university-educated participants expressing greater interest in interventions related to mood (*P*=.01), socialization (*P*=.02), memory (*P*=.01), and computer-based exercises (*P*=.046). eHealth preferences also varied in association, with diagnosis for interventions targeting sleep (*P*=.01), nutrition (*P*=.004), vascular risk factors (*P*=.03), and memory (*P*=.02).

**Conclusions:**

Technology use is pervasive among older adults with cognitive impairment, though variability was noted in relation to age, education, vocational status, and diagnosis. There is also significant interest in Web-based interventions targeting cognition and memory, as well as other risk factors for cognitive decline, highlighting the urgent need for the development, implementation, and study of eHealth technologies tailored specifically to older adults, including those with MCI and early dementia. Strategies to promote eHealth use among older adults who are retired or have lower levels of education will also need to be considered.

## Introduction

It is estimated that by 2050 there will be over 115 million people living with dementia worldwide [1]. As there are currently no cures for dementia, efforts are increasingly focused on targeting potentially modifiable risk factors for cognitive decline [2-4], with particular emphasis on intervention early in the disease course [5]. Recent meta-analytic data highlight that approximately one-third of the burden of Alzheimer’s disease can be attributed to seven key modifiable risk factors, including depression, diabetes, midlife hypertension, midlife obesity, smoking status, low physical activity, and low educational attainment [6]. In turn, it is estimated that a mere 10% reduction per decade for each of these modifiable risk factors could reduce the prevalence of Alzheimer’s disease by 8.3% in 2050 [6]. Although prevention is the ultimate goal, supportive programs for individuals experiencing cognitive decline or dementia and their carers are also essential to reduce the risk of further cognitive decline, medical comorbidities, mental health problems, and functional decline, as well as to promote quality of life, healthy brain aging, and general well-being. Given the scale of the dementia health care crisis globally, low-cost, effective, and easily accessible strategies addressing these modifiable risk factors and providing support for people with dementia are required. In this regard, there is increasing interest in the use of Internet technologies, particularly electronic health (eHealth).

Increasingly, the Internet is becoming a critical medium for the delivery of medical and mental health information and services, referred to as *eHealth*. eHealth is broadly defined by the World Health Organization as the use of information and communication technologies for health-related purposes such as service delivery [7]. eHealth tools such as mobile and Internet-based apps can be used to screen “at risk” individuals, offer self-help through Web-based interventions, or deliver proactive and guided interventions. eHealth interventions have been shown to be effective for the management and/or treatment of symptoms in a range of mental health and medical conditions, including depression [8-10], diabetes [11], weight loss [12], problematic alcohol use [13], sleep [14], and exercise [15]. Various models of eHealth services have been shown to be successful, including stand-alone systems for symptom prevention and self-help, consumer-assisted care such as peer support, virtual clinics offering professional care, and stepped care systems for integrated care [16]. There is also an emerging literature regarding important methodological considerations affecting adherence (eg, interface design and feasibility testing) and treatment outcomes (eg, time spent in activities) [17-20].

The growing interest in the utility of mobile and Internet-based apps and e-tools for health-related purposes has been facilitated by a dramatic increase over the last two decades in Internet access worldwide. As of 2016, 40% of the global population had an Internet connection compared with only 1% in 1995 [21]. In relation to specific regions, there has been a 500% growth in Internet usage in Europe from 2000 to 2017, with 77% of the population now having access [22]. Similarly, 88% of the population in North America had Internet access in 2017, reflecting an almost 200% increase since 2000 [22]. Importantly, older adults represent the fastest growing group of Internet users [23]. This increase in Internet use among this population has spurred a growing interest in the development and implementation of eHealth technologies for improved health and well-being for older adults [24-26].

However, to date, there has been limited research evaluating the utility of eHealth technologies for the prevention or slowing of cognitive decline in older adults. Additionally, there are no published studies specifically targeting people with existing cognitive impairment, such as mild cognitive impairment (MCI). This represents a significant gap, given that approximately 45% of people with MCI convert to dementia within 5 years [27] and that secondary prevention strategies for cognitive decline are likely to be optimal during this critical period [5,28-32]. Such technologies could be employed for information provision, for interventions encouraging social engagement, physical or cognitive exercise, for treating depression and sleep, and for provision of adaptive or compensatory strategies to improve memory or daily functioning [29]. Importantly, one small study of 37 people with MCI demonstrated that participants utilized the Internet to the same extent as cognitively intact older people, with 73% of those with MCI using such technologies to search for health care–related information and 81% reporting technology use for communication [33]. Despite these promising figures, older people do have more difficulty engaging with the Internet for health care [34], which has been attributed, at least in part, to poor website design, complex navigation requirements, and a lack of Internet training—factors that are secondary to cognitive decline and can be addressed with further research [35].

Whereas mobile and Internet-based apps and e-tools hold great promise in relation to the promotion of healthy aging and the self-management of health-related conditions and modifiable risk factors of cognitive decline, it is first necessary to better understand the feasibility and likely acceptability of such e-tools for older adults. Therefore, this study was designed to characterize the current patterns of Internet use, as well as interest in eHealth technologies (ie, mobile- and Internet-based apps and e-tools) among older adults with varying degrees of cognitive impairment ranging from subjective cognitive complaints to MCI and dementia. We also aimed to generate prevalence data essential to determining the feasibility of future eHealth efforts in an aging population.

## Methods

### Participants

From February 2015 to October 2016, data were collected from the Healthy Brain Ageing (HBA) Clinic cohort at the Brain and Mind Centre, University of Sydney, Sydney, Australia. Participants attending the HBA Clinic, an early intervention clinic for people aged 50 years or older, represent an inner-city cross section of the population. They were all asked to complete a self-report survey regarding patterns of Internet use, as well as interest in and preferences for eHealth technologies, including mobile and Web-based interventions targeting individual risk factors for cognitive decline and dementia. This patient population was specifically chosen to evaluate the potential to use eHealth technologies with older adults with cognitive impairment or early dementia.

Consecutive referrals of adults were invited to participate. Exclusion criteria included limited English proficiency, intellectual disability, Mini-Mental State Examination <20 (MMSE; [36]), history of stroke, traumatic brain injury (with loss of consciousness >30 min), neurological or other medical conditions known to affect cognition, and current substance misuse or major psychiatric disorder (eg, psychosis). All participants were referred to the HBA Clinic by their general practitioner or specialist clinician for evaluation because of concerns regarding their cognition or mood. Inclusion and exclusion criteria were verified with participants over the phone by a member of the research team before completing the face-to-face medical, neuropsychological, and mood assessments.

### Assessments

#### Diagnosis and Clinical Characteristics

As described previously (Jayaweera et al [37]), all eligible participants underwent a comprehensive clinical assessment. A specialist physician (geriatrician or neurologist) carried out a structured review of medical and psychiatric history, and a clinical neuropsychologist administered a standardized neuropsychological evaluation. Participants with no evidence of objective cognitive impairment were classified as having subjective cognitive impairment (SCI). Diagnoses of MCI and early dementia were determined by consensus rating of 3 raters, including a neurologist or geriatrician and 2 clinical neuropsychologists. Background and medical history, clinical presentation, neuropsychological performance, and neuroimaging findings (if available) were all taken into account in the diagnostic process. Using established criteria [38], MCI was defined as at least a 1.5 standard deviation decline on one or more neuropsychological tests relative to the participant’s estimated baseline level of performance, alongside subjective complaints and in the absence of significant functional decline. Established diagnostic criteria were also utilized in the differential diagnosis of dementia [39-41].

#### HBA eHealth Questionnaire

Each participant completed the HBA E-Health Questionnaire (see Multimedia Appendix 1), a 20-item self-report survey designed by members of the HBA team at the University of Sydney, to identify patterns of technology and Internet use in older people, with an emphasis on the current use of or interest in health-related e-tools. For example, questions included “Do you have access to the Internet at home?” and “Would you use the Internet to receive programs or interventions for any of the following: mood, sleep, exercise, nutrition, socialization, management of vascular risk factors, practical strategies for memory, or online computer exercise for cognition?” The survey was created to inform the development, feasibility, acceptability, and delivery of future eHealth trials with older adults. This is a newly developed measure that has not been used in previous research studies.

Importantly, during data collection, we identified several additional issues related to technology use that we believed were relevant in relation to eHealth practices of older adults. As such, the HBA E-health Questionnaire was revised, accounting for the variability in the number of respondents for some questions. The second version of the questionnaire included more specific questions about how individuals connect to the Internet, website preferences, confidence in the information available on health-related websites, and barriers to accessing information on health-related websites.

### Data Collection

After being scheduled to attend the HBA Clinic, printed questionnaires were sent by mail to verbally consenting participants, along with detailed study information and a consent form. Some participants may have received documents by email at their request. Participants had the option to return completed questionnaires by mail (postage paid) or to bring questionnaires with them to their HBA Clinic appointment for collection by research staff. All questionnaires were handled by members of the HBA Clinic and were briefly reviewed for missing items by the clinic coordinator on participant arrival to their clinic appointment. Missing responses were subsequently collected from the participant in person or via telephone.

### Data Analysis

Descriptive statistics were used to analyze all aspects of the survey data. Given that the overall sample size was <300 and that the subset of participants with early dementia was relatively small (n=23), bivariate analyses using Fisher exact tests were used to evaluate group differences. To determine the association between sociodemographic factors, including age, years of education, gender, vocational status, and diagnosis on eHealth preferences, binary logistic regression models were constructed with all variables entered into the model in block 1 (method: enter). All of the assumptions of binary logistic regression were examined and met. The alpha level was <.05. The Statistical Package for the Social Sciences (SPSS) version 24 (IBM Corp) was used for all analyses.

### Ethics Approval and Registration

Participation was voluntary, and written informed consent was obtained from all participants. Ethical approval was obtained from the University of Sydney Human Research Ethics Committee (Project number: 2012/1873).

## Results

### Participants

A total of 221 participants (mean age=67.6 years, range=51-88 years; 57.5% [127/221] female; and mean MMSE=28.2, range=20-30) from the HBA Clinic completed the survey. Three participants who provided written consent to the undergo medical, neuropsychological, and mood assessments at the HBA Clinic failed to complete the self-report questionnaire. Demographic characteristics of the participants are presented in Table 1. Notably, participants had above average levels of education, and the majority were retired (142/218, 65.1%).

### Computer Use

The majority of participants (167/194, 86.1%) reported using a computer routinely, defined as more than 4 times a week. Most respondents had access to an electronic device at home, primarily in the form of a computer (205/221, 92.8%), though more than one-third of respondents also had access to a tablet (87/221, 39.4%). Only 5 participants had no access to a computer or tablet (5/221, 2.3%). There was no notable difference in the prevalence of computer use across gender (*P*=.57). Whereas, as noted above, the overwhelming majority of participants used computers, responses indicated that older participants (≥65 years) were significantly less likely to use a computer relative to middle-aged respondents (50-64 years) (*P*<.001; Table 2). Similarly, markedly, fewer respondents with lower levels of education (less than a bachelor’s degree) reported using a computer relative to those with at least a university degree (*P*<.001; Table 2).

**Table 1:** Demographic characteristics of study participants.

Characteristic			Descriptive statistic
**Continuous variables**			
	Age, in years **,** mean (SD)		67.6 (8.5)
	Years of education, mean (SD)		14.0 (3.1)
	MMSE^a^, mean (SD)		28.2 (2.0)
**Categorical variables**			
	**Gender**		
		Female, n (%)	127 (57.5)
		Male, n (%)	94 (42.5)
	**Vocational status**		
		Retired, n (%)	142 (65.1)
		Full-time employment, n (%)	31 (14.2)
		Part-time employment, n (%)	31 (14.2)
		Other^b^, n (%)	14 (6.5)
	**Diagnosis**		
		Mild cognitive impairment, n (%)	137 (62.0)
		Dementia^c^, n (%)	23 (10.4)
		Subjective cognitive complaints only, n (%)	61 (27.6)

^a^MMSE: Mini-Mental State Examination.

^b^Includes individuals who are homemakers (3/14, 20%), full-time students (2/14, 13%), on medical or psychiatric leave of absence (1/14, 6%), discontinued work or study because of illness (1/14, 6%), currently unemployed (5/14, 36%), or other (2/14, 13%).

^c^Dementia diagnoses include Alzheimer’s disease (20/23, 87%), fronto-temporal dementia (1/23, 3%), and mixed dementia or unknown etiology (2/23, 9%).

**Table 2:** Frequency of use of computers, mobile phones, and health-related websites. Discrepancies in the number of respondents for some questions relate to an update to the questionnaire during the data collection process (refer to Methods).

Sociodemographic variable		Computer use: yes		Smartphone: yes		Texting: yes		Use health-related websites: yes	
		n (%)	*P* value	n (%)	*P* value	n (%)	*P* value	n (%)	*P* value
**Age group, in years**			<.001		.001		<.001		.01
	50-64	77 (95)		52 (65)		75 (96)		59 (77)	
	≥65	115 (81.6)		65 (46.4)		99 (76.7)		88 (59.9)	
**Level of education**			<.001		.002		.10		.004
	<Bachelor’s degree	87 (79.1)		47 (43.1)		83 (81.4)		65 (56.9)	
	≥Bachelor’s degree	103 (94.5)		69 (63.3)		90 (88.2)		81 (75.6)	
**Vocational status**			.009		.007		<.001		.01
	Working^a^	59 (94)		40 (65)		58 (97)		48 (80)	
	Retired	119 (83.8)		66 (46.5)		101 (77.7)		88 (60.3)	
	Other^b^	11 (79)		9 (68)		13 (93)		10 (63)	
**Diagnosis**			<.001		.001		<.001		.001
	SCI^c^	57 (92)		35 (57)		55 (95)		40 (68)	
	MCI^d^	120 (87.6)		76 (55.5)		106 (83.5)		96 (70.6)	
	Dementia	15 (64)		6 (25)		13 (62)		9 (31)	

^a^Part- or full-time gainful employment.

^b^Includes individuals who are homemakers (3/14, 20%), full-time students (2/14, 13%), on medical or psychiatric leave of absence (1/14, 6%), discontinued work or study because of illness (1/14, 6%), currently unemployed (5/14, 36%), or other (2/14, 13%).

^c^SCI: subjective cognitive impairment.

^d^MCI: mild cognitive impairment.

Also shown in Table 2, working participants were more likely to use a computer as opposed to those who were retired or otherwise not formally employed (*P*=.009) Computer use also varied by degree of cognitive impairment (*P*<.001), and the means suggest that participants who met the criteria for early dementia were less likely to use a computer than both individuals with SCI and those with MCI (Table 2).

### Mobile Phone Use

The vast majority of participants reported having a mobile phone (201/220, 91.4%), with approximately half using a smartphone (117/220, 53.2%). Most respondents already used texting (174/206, 84.5%), and a small group preferred to access the Internet via their mobile phone (18/183, 9.8%). Mobile phone use did not differ by gender (*P*=.83); however, as shown in Table 2, the middle-aged participants were significantly more likely to have a smartphone (*P*=.001) and to use texting relative to older respondents (*P*<.001). Although more university-educated participants reported having smartphones (*P*=.002), there was no difference in the use of texting compared with respondents with fewer years of education (*P*=.10; Table 2). Retired adults were also less likely to have a smartphone (*P=*.007) or to use texting (*P<*.001). Again, the proportion of participants who had a smartphone (*P*=.001) and who used texting (*P*<.001) varied with diagnosis, with respondents with early dementia appearing to be less likely to use either compared with those with SCI or MCI (Table 2).

### Internet Practice

#### Access

The overwhelming majority of participants had access to the Internet at home (204/220, 92.6%), primarily via a computer. Approximately three-quarters of respondents reported using the Internet without difficulty (164/220, 74.5%), whereas a very small portion of the sample indicated that they lacked the skills to use the Internet proficiently (14/220, 6.4%). Internet use did not differ markedly across gender (*P*=.31); however, respondents over 65 years of age (23/140, 16.3%) or with lower levels of education (19/109, 17.4%) were more likely to experience difficulties, need assistance, or lack the skills required to use the Internet reliably relative to middle-aged participants (2/80, 3%; *P*<.001) or those who were more educated (6/109, 5.5%; *P*=.006). Importantly, participants who were retired (98/141, 69.5%) or otherwise not engaged in gainful employment (10/14, 70%) were equally able (*P*=.07) to use the Internet without complications relative to employed participants (53/62, 86%). Similarly, respondents with early dementia were not more likely (*P*=.08) to experience difficulties using the Internet (4/23, 16%) relative to those with SCI (4/61, 7%) or MCI (17/136, 12.5%). The majority of participants used a broadband or digital subscriber line connection at home to access the Internet (115/183, 62.8%), and most respondents were satisfied with the speed of their Internet connection (151/176, 85.8%).

#### Internet Activities

Most participants used email (196/217, 90.3%); however, the use of social media websites was less common (Facebook: 93/216, 43.1%; Twitter: 12/216, 5.6%; Instagram: 15/216, 6.9%; Pinterest: 20/216, 9.3%; and LinkedIn: 40/216, 18.5% or 30/122, 24.6% of working respondents). Furthermore, the majority of respondents indicated that they used the Internet most frequently for email relative to other common Web-based activities, including social connectedness, searching for information, and reading the news. Of note, older respondents were significantly less likely to use Facebook relative to middle-aged respondents (*P*=.002). Additionally, diagnosis (*P*=.02) was associated with the use of Facebook, with the percentages suggesting that individuals with early dementia (6/27, 21%) are less likely to use Facebook relative to those with SCI (32/62, 52%) or MCI (58/141, 41%).

#### eHealth Engagement

As shown in Table 2, the reported use of health-related websites varied considerably. A small proportion of the participants reported regular use of health-related websites, and approximately half of the respondents visited health-related websites occasionally. However, one-fifth of the sample (42/207, 20.3%) indicated they would never use health-related websites, primarily because of a lack of interest (42/112, 37.5%). Strikingly, the vast majority of participants who access health information via the Internet are at least reasonably confident in the reliability of information (reasonably confident: 62/114, 54.4%; quite confident: 18/114, 15.8%; and very confident: 5/114, 4.4%). As mentioned in the Methods section, discrepancies in the number of respondents for the latter questions relates to an update to the questionnaire during the data collection process. As shown in Table 2, the use of health-related websites did not differ by gender (*P*=.78) but varied with age (*P*=.01), education (*P*=.004), vocational status (*P*=.01), and diagnosis (*P*=.001).

### Interest In and Preferences for eHealth Technologies

**Figure 1:**
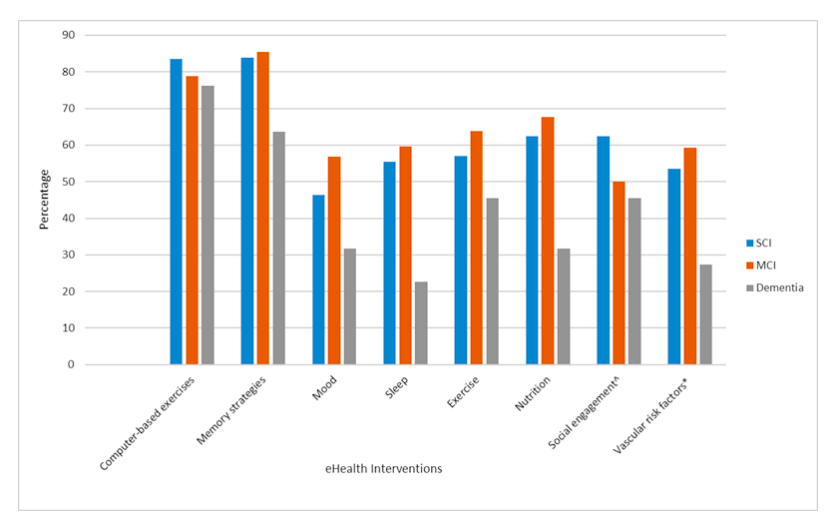
Interest in electronic health (eHealth) technologies varies with diagnosis. Abbreviations: SCI: subjective cognitive impairment, MCI: mild cognitive impairment, ^eg, outings, public talks and seminars, groups, etc, and *eg, high blood pressure, cholesterol, etc.

The majority of participants (198/209, 94.7%) reported that they would find it useful to be able to access a website designed to support healthy aging, including physical health and cognition, self-manage existing conditions, and track changes in cognition over time. Similarly, most respondents also reported interest in a website designed to specifically measure mood-related concerns and changes (172/206, 83.5%). When asked about Web-based interventions targeting individual risk factors for cognitive decline and dementia, there was an overwhelming interest in programs offering practical memory strategies and computer exercises to improve cognition (see Figure 1). Although not as pronounced, there was also considerable interest in Web-based interventions for a range of health concerns and lifestyle factors, including mood, sleep, physical activity, diet and nutrition, social engagement, and the management of vascular risk factors. Notably, preferences for eHealth technologies did not differ on the basis of employment status. Similarly, interest in eHealth interventions generally did not differ in relation to age and gender. However, middle-aged participants were more interested in interventions for sleep (*P*=.005) relative to the older respondents, and women were more interested in social programs (*P*=.004) compared with men. In relation to education, university-educated participants expressed greater interest in interventions relating to mood (*P*=.01), socialization (*P*=.02), memory (*P*=.014), and computer-based exercises (*P*=.046) compared with those with fewer years of education. Finally, variability in the preference for eHealth technologies varied in association with diagnosis for interventions targeting to sleep (*P*=.01), nutrition (*P*=.004), vascular risk factors (*P*=.03), and memory (*P*=.02). As presented in Figure 1, it appears that individuals with SCI and MCI were more likely to indicate interest in the aforementioned interventions relative to those with early dementia. In general, participants with MCI were most likely to indicate that they would use Web-based interventions.

After confirming that all assumptions had been met, age, years of education, gender, vocational status, and diagnosis were entered into a binary logistic regression to determine their association with interest in eHealth interventions for memory. As shown in Table 3, the model was statistically significant (χ^2^_7_=19.1, *P*=.008), explaining 13.8% of the variance in the preference for interventions targeting memory. Younger age (*P*=.02), more years of education (*P*=.03), and being retired (*P*=.03) were associated with a greater likelihood of being interested in eHealth interventions for memory, whereas those participants with a diagnosis of dementia were significantly less likely to be interested in such interventions relative to those with SC1 or MCI (*P=*.02).

As shown in Table 4, a similar model was generated to evaluate the relationship between sociodemographic factors and a preference for eHealth interventions targeting sleep. Again, the model was statistically significant (χ^2^_7_=22.7, *P*=.002). The model explained 13.3% of the variance in the preference for sleep-related interventions, with younger age (*P*=.001) and a diagnosis of dementia being the significant predictors (*P*=.008).

**Table 3:** Predictors of interest in electronic health (eHealth) interventions for memory.

Variables		Beta	SE^a^	Wald test	*P* value	Odds ratio (95% CI)
Age		−.06	0.03	4.72	.02	0.93 (0.88-0.98)
Gender (female)		−.03	0.40	0.01	.91	0.95 (0.43-2.10)
Years of education		.13	0.05	4.87	.03	1.14 (1.02-1.31)
**Vocational group**						
	Working^b^			4.55	.09	
	Retired	−1.32	0.92	1.98	.16	0.27 (0.03-1.67)
	Other^c^	−.18	0.87	0.04	.81	0.81 (0.14-4.50)
**Diagnosis**						
	SCI^d^			6.94	.02	
	MCI^e^	.20	0.46	0.20	.64	1.24 (0.49-3.12)
	Dementia	−1.19	0.48	5.83	.02	0.31 (0.12-0.79)

^a^SE: standard error.

^b^Part- or full-time gainful employment.

^c^Includes individuals who are homemakers (3/14, 20%), full-time students (2/14, 13%), on medical or psychiatric leave of absence (1/14, 6%), discontinued work or study because of illness (1/14, 6%), currently unemployed (5/14, 36%), or other (2/14, 13%).

^d^SCI: subjective cognitive impairment.

^e^MCI: mild cognitive impairment.

**Table 4:** Predictors of interest in electronic health (eHealth) interventions for sleep.

Variables		Beta	SE^a^	Wald test	*P* value	Odds ratio (95% CI)
Age		−.06	0.01	10.87	.001	0.92 (0.88-0.98)
Gender (female)		.08	0.29	0.10	.76	1.10 (0.61-1.97)
Years of education		−.003	0.05	0.004	.95	1.00 (0.91-1.10)
**Vocational group**						
	Working^b^			2.45	.28	
	Retired	−.73	0.67	1.17	.28	0.47 (0.12-1.78)
	Other^c^	−.13	0.63	0.03	.83	0.87 (0.24-3.07)
**Diagnosis**						
	SCI^d^			7.17	.03	
	MCI^e^	−.29	0.33	0.80	.36	0.74 (0.38-1.44)
	Dementia	−1.26	0.48	7.03	.008	0.27 (0.10-0.72)

^a^SE: standard error.

^b^Part- or full-time gainful employment.

^c^Includes individuals who are homemakers (3/14, 20%), full-time students (2/14, 13%), on medical or psychiatric leave of absence (1/14, 6%), discontinued work or study because of illness (1/14, 6%), currently unemployed (5/14, 36%), or other (2/14, 13%).

^d^SCI: subjective cognitive impairment.

^e^MCI: mild cognitive impairment.

Another statistically significant model (χ^2^_7_=19.9, *P*=.005) explained 11.8% of the variance in the interest in social eHealth programs, indicating that being female (*P*=.001) and having more years of education (*P*=.01) were both significantly associated with a preference for this type of intervention (Table 5).

As displayed in Table 6, the same sociodemographic factors were entered into a logistic regression model to examine their relationship with interest in eHealth interventions targeting mood. The model was statistically significant (χ^2^_7_=14.1, *P*=.047). The model explained 8.5% of the variance in the preference for interventions for mood, with younger age (*P*=.011) being the only significant predictor.

Interest in eHealth interventions for nutrition was also significantly associated with sociodemographic factors (χ^2^_7_=21.0, *P*=.004). As shown in Table 7, the model explained 12.5% of the variance in preference for nutrition interventions. Younger participants were significantly more likely to be interested (*P=*.01), whereas participants with dementia were significantly less likely to endorse this preference (*P=*.001).

On the basis of binary logistic regression models, no significant associations were found between the aforementioned sociodemographic variables and a preference for eHealth interventions targeting exercise (*P*=.08), vascular risk factors (*P*=.08), and computer-based exercises (*P*=.12).

**Table 5:** Predictors of interest in electronic health (eHealth) interventions for socialization.

Variables		Beta	SE^a^	Wald test	*P* value	Odds ratio (95% CI)
Age		−.02	0.01	2.51	.10	0.97 (0.93-1.01)
Gender (female)		1.00	0.29	11.10	.001	2.72 (1.50-4.94)
Years of education		.11	0.05	6.01	.01	1.13 (1.03-1.23)
**Vocational group**						
	Working^b^			3.00	.21	
	Retired	−.61	0.60	1.03	.30	0.54 (0.15-1.79)
	Other^c^	.08	0.57	0.02	.88	1.07 (0.33-3.37)
**Diagnosis**						
	SCI^d^			2.26	.31	
	MCI^e^	.48	0.33	2.03	.14	1.64 (0.82-3.20)
	Dementia	−.06	0.44	0.03	.87	0.93 (0.38-2.18)

^a^SE: standard error.

^b^Part- or full-time gainful employment.

^c^Includes individuals who are homemakers (3/14, 20%), full-time students (2/14, 13%), on medical or psychiatric leave of absence (1/14, 6%), discontinued work or study because of illness (1/14, 6%), currently unemployed (5/14, 36%), or other (2/14, 13%).

^d^SCI: subjective cognitive impairment.

^e^MCI: mild cognitive impairment.

**Table 6:** Predictors of interest in electronic health (eHealth) interventions for mood.

Variables		Beta	SE^a^	Wald test	*P* value	Odds ratio (95% CI)
Age		−.04	0.01	6.40	.01	0.95 (0.90-0.99)
Gender (female)		.29	0.28	0.96	.31	1.32 (0.74-2.36)
Years of education		.03	0.05	0.35	.54	1.03 (0.94-1.13)
**Vocational group**						
	Working^b^			0.69	.69	
	Retired	−.48	0.61	0.57	.45	0.61 (0.17-2.10)
	Other^c^	.22	0.60	0.14	.70	0.78 (0.25-2.54)
**Diagnosis**						
	SCI^d^			4.64	.10	
	MCI^e^	−.57	0.32	3.05	.79	0.56 (0.29-1.06)
	Dementia	−.70	0.44	2.51	.10	0.48 (0.19-1.17)

^a^SE: standard error.

^b^Part- or full-time gainful employment.

^c^Includes individuals who are homemakers (3/14, 20%), full-time students (2/14, 13%), on medical or psychiatric leave of absence (1/14, 6%), discontinued work or study because of illness (1/14, 6%), currently unemployed (5/14, 36%), or other (2/14, 13%).

^d^SCI: subjective cognitive impairment.

^e^MCI: mild cognitive impairment.

**Table 7:** Predictors of interest in electronic health (eHealth) interventions for nutrition.

Variables		Beta	SE^a^	Wald test	*P* value	Odds ratio (95% CI)
Age		−.06	0.01	6.81	.01	0.93 (0.89-0.97)
Gender (female)		.40	0.29	1.83	.18	1.50 (0.82-2.74)
Years of education		−.011	0.04	0.04	.81	0.99 (0.90-1.08)
**Vocational group**						
	Working^b^			4.64	.10	
	Retired	−.68	0.65	1.10	.30	0.49 (0.14-1.83)
	Other^c^	.19	0.64	0.09	.74	1.23 (0.34-4.27)
**Diagnosis**						
	SCI^d^			10.52	.01	
	MCI^e^	−.23	0.35	0.48	.49	0.79 (0.40-1.54)
	Dementia	−1.50	0.47	10.52	.001	0.21 (0.09-0.55)

^a^SE: standard error.

^b^Part- or full-time gainful employment.

^c^Includes individuals who are homemakers (3/14, 20%), full-time students (2/14, 13%), on medical or psychiatric leave of absence (1/14, 6%), discontinued work or study because of illness (1/14, 6%), currently unemployed (5/14, 36%), or other (2/14, 13%).

^d^SCI: subjective cognitive impairment.

^e^MCI: mild cognitive impairment.

## Discussion

### Principal Findings

Our results demonstrate that technology use is pervasive among older adults presenting to a specialized metropolitan early intervention clinic for cognition and mood in an Australian context. Specifically, the data show that 91.4% (201/220) of participants used a mobile phone, with 53.2% (117/220) using a smartphone, and 92.8% (205/221) had access to a computer in the home, with 39.4% (87/221) of participants also using a tablet. Whereas computer use varied somewhat based on age, education, vocational status, and diagnosis, the vast majority of participants used computers routinely irrespective of these factors. Importantly, this is the first study to highlight that older adults with cognitive impairments that may affect Internet and mobile phone use are still actively engaging with technology.

In accordance with global data showing growing computer use and Internet access among older adults, a striking 92.7% (204/220) of our respondents have access to the Internet at home. Adults ≥65 years and with fewer years of schooling were more likely to require assistance, experience difficulties, or lack the necessary skills to use the Internet relative to participants aged 50 to 64 years and those with higher levels of education. Importantly, however, diagnosis did not impact upon proficiency in Internet use. Of significance, the prevalence of Internet access and use within this study sample exceeds the 2015 estimates reported by the Australian Bureau of Statistics [42]. This may relate to sociodemographic factors specific to the study sample, including having above average levels of education and residing in a metropolitan area. Indeed, it is likely that in rural and remote regions, Internet access may not be as readily available [43]. Similarly, older age and lower socioeconomic status are also associated with lower rates of Internet access [44]. Of note, residents of Greater Sydney, which would mostly comprise our study participants, report higher wages and total annual income relative to other regions in the state [45].

The finding that older people with MCI or early dementia have access to technology and the Internet indicates that targeted eHealth interventions could be developed to address modifiable risk factors. A recent study reported that 63.1% of 1014 community-dwelling older adults aged 57 to 77 years would use eHealth if given the opportunity [46]. This is consistent with our finding of 67.6% (140/207) of respondents visiting health-related websites either regularly or occasionally (see Table 2). Our results further highlight that individuals with MCI and early dementia are also interested in using eHealth interventions for cognition, lifestyle factors, and health concerns, suggesting the potential for the targeted use of eHealth technology in these groups. However, this study is the first to show that preferences for eHealth differ depending on the severity of cognitive impairment. Whereas interest in computer-based cognitive exercises was roughly equivalent across diagnostic groups, those with SCI and MCI expressed greater interest (>80% of those groups) in Web-based strategies specifically targeting memory relative to respondents with early dementia. Additionally, younger age and higher levels of education were also associated with an increased preference for memory-related eHealth interventions. These group differences may reflect the health-seeking status of participants with SCI and MCI, as well as the concomitant desire to delay or prevent cognitive decline among middle-aged educated adults. Notably, however, 63.6% (14/22) of individuals with early dementia were also interested in Web-based memory activities.

In relation to other eHealth interventions, participants with early dementia appeared most interested in those designed to facilitate exercise, as well as to improve social engagement and participation; however, given the small sample size of this subgroup, these data are interpreted cautiously. With this caveat in mind, the relative interest in social programs may reflect the isolation that often occurs with aging and in particular, when an individual is diagnosed with dementia [47]. A longitudinal cohort study of >4000 older adults reported that the combination of Internet use and social engagement (eg, attending art exhibits, movies, and theatre) appeared to help older adults maintain the health literacy skills necessary to manage their health, including the ability to understand basic health information and services [48]. Similarly, it has been shown that Internet use for communication and social support is associated with enhanced life satisfaction, psychological well-being, and sense of community [49,50].

Social media networks also have the potential to promote socialization among older adults, regardless of geographic location and mobility issues. Of commonly used social networks, our results demonstrate that older adults are most likely to use Facebook, which has specifically been shown to be associated with social connectedness and well-being in older adults [51] and may have the potential to improve executive functions and processing speed [52]. That being said, in our sample, the older participants (aged ˃65 years) were significantly less likely to use Facebook relative to the middle-aged respondents. Additionally, our results show that men are less interested in social eHealth programs. Despite potential benefits, adults in later life may have negative attitudes toward social media for varying reasons, including concerns regarding data privacy, a lack of familiarity with Web-based social norms, and discomfort with self-disclosure [53]. Therefore, it is recommended that novice users, which may include more men than woman, are supported by a moderator to help them overcome potential barriers. In addition, the rates of social media use among this well-educated sample were relatively low. Thus, given the potential benefits of engaging with social media networks, future efforts to promote the use and uptake of social media would be vital for programs or interventions that target older adults with concerns about their cognition.

With regard to exercise, recent meta-analytic data showed that exercise is beneficial for cognition in people with dementia [54], more so than other nonpharmacological interventions such as music therapy and cognitive training [55]. Web-based interventions have already proved effective as a method to promote exercise in older adults [15], particularly when they take into account environmental factors such as local neighborhood offerings for physical activity and are tailored to older adults, with the potential to be personalized and adapted to each individual [25]. In light of the existing literature and the relative interest in exercise programs reported in our sample, feasibility and efficacy studies of Web-based exercise interventions for people with dementia are now essential.

Interestingly, a relatively higher percentage of respondents with SCI and MCI generally reported an interest in interventions addressing sleep, nutrition, and vascular risk factors, relative to those with early dementia. Importantly, there is an extensive literature highlighting the benefits of early intervention for cognitive decline [5,28-32]. Indeed, our prior trials of healthy brain-aging cognitive training have been successful and have been shown to improve knowledge, memory, mood, and sleep, as well as reduce disability in people with neurodegenerative diseases and depression [31,56-58].

Studies investigating the potential for targeted interventions of this sort to be delivered via the Internet are now required. In this regard, there is a growing literature regarding the use of eHealth interventions for a range of medical and mental health conditions, including modifiable risk factors for cognitive decline [8-13,15]. However, at present, there are no known eHealth interventions specifically for people with MCI or dementia. There are, however, several large-scale clinical trials in various stages of completion seeking to evaluate the utility of Web-based lifestyle interventions for older adults. For example, Glozier et al [10] showed that an Internet-based cognitive behavioral therapy (CBT) intervention resulted in a significant decrease in depressive symptoms in people with mild-to-moderate depression and high levels of cardiovascular risk factors. There is also evidence to support the use of a Web-based CBT insomnia program (ie, SHUT-I) for the treatment of depression in adults over the age of 50 years [14], including forthcoming data from the Sleep or Mood Novel Adjunctive Therapy trial (ANZCTR12612000985886) [59]. The Body, Brain, Life program (ANZCTR12612000147886), a 12-week dementia risk reduction intervention, was shown to result in a significant decrease in dementia risk among cognitively intact adults (n=58) aged 50 to 60 years at 26 weeks, largely because of an increase in positive protective factors such as fish consumption and cognitive engagement [60]. Similarly, the Maintain Your Brain trial aims to recruit 18,000 people to evaluate the benefits of Internet coaching on dementia risk [61].

As eHealth interventions and clinical registries are being developed and tailored specifically for individuals with MCI and early dementia, it will be essential to investigate potential predictors of use such as level of education, vocational status, degree of cognitive impairment, and medical burden. The optimal timing, frequency, and intensity, as well as the method of delivery (eg, via mobile phone, computer, or tablet) of the intervention may also impact on the acceptability and feasibility of eHealth tools. Given the common use of texting in our sample (174/206, 84.5%), texts may be an easy and cost-effective way in which to provide reminders and key tips and suggestions. Responsive websites that are mobile-friendly and can adapt to any sized device will offer broad accessibility; however, apps allow for personalization of the features and are preferred for interactive games. Our data suggest that adults aged 65 years and older would be more likely to utilize computer-based interventions; however, given that 65% (80/220) of younger (50-64 years) respondents had a smartphone, they may be more apt to use mobile apps, allowing for push notifications, data tracking, and social sharing of content. Importantly, apps also allow content to be downloaded so that it can be accessed without an Internet connection, which may be particularly important in areas with limited and/or unreliable Internet service.

The efficacy of eHealth interventions may vary in relation to the provision of supervision by a coach or clinician [62], as well as severity of the impairment in the target population [29]. In relation to the latter, future eHealth interventions should offer hierarchical support, adapting to the ability level of the patient and guiding them in the selection of the most appropriate intervention given their level of impairment, as well as personal preferences. Factors relating to adherence, including clinician support and coordination of clinical care with eHealth [17], will also need to be further explored. Additionally, researchers will also need to carefully consider recruitment methods when evaluating such interventions to avoid selection bias. Whereas social media networks such as Facebook are optimal for recruiting participants for eHealth studies, the use of such sites differs notably by age [63]. Additionally, although Facebook is the most used site irrespective of age [63], as it was with our participants, a recent study showed that recruitment rates and participant engagement varied based on the content of Facebook advertisements, impacting upon the generalizability of the results [64]. Therefore, recruitment methods will need to be carefully tailored to the target audience.

### Limitations

Ultimately, eHealth technologies offer a unique opportunity for scalable and cost-effective screening of cognition and modifiable risk factors of cognitive decline linked with evidence-based, multidisciplinary interventions in a systematic and stepwise fashion, with the primary aim of improving the accessibility of individualized care for older adults. This study is the first to examine computer, Internet, mobile phone, and eHealth technology use with regard to cognitive status in older adults. However, it is important to interpret our findings in the context of study limitations. The reliability of information gathered via self-report questionnaire may be reduced in people with dementia, depending on their degree of cognitive impairment. We must also acknowledge that the study sample may not be representative of the broader population because of socioeconomic factors, including years of education (mean=14.0 years), residency in a metropolitan area, and annual income. It will be important in the future to gather the same type of prevalence data in regional and remote settings, particularly as it is in these settings where eHealth technologies may have a greater impact by increasing access to care. In the future, we propose to update the HBA E-health Questionnaire to broaden the definition of texting to include other communication methods such as Messenger and WhatsApp developed by Facebook, and Viber developed by Rakuten Inc. We will also include additional questions about the use of tablets (ie, iPad, Apple Inc) to determine the appropriateness of eHealth interventions delivered in this format. This is of particular interest as many existing Web-based cognitive tests (ie, Cambridge Neuropsychological Test Automated Battery [65]) are being developed for tablets and, therefore, would provide an opportunity to assess and track cognitive performance in older adults over time and in conjunction with specific eHealth interventions. It is also important to consider that the phrasing of some questions in the survey (eg, “Would you use...”) may have prompted positive responses from respondents, potentially biasing the results. However, approximately 40% to 50% of participants indicated that they were, in fact, *not* interested in several of the interventions, arguing against this concern.

### Conclusions

This study presents key data showing the use of and interest in eHealth technologies in older Australian adults with cognitive impairment. Overall, our data demonstrate an overwhelming interest within this demographic for targeted interventions to address modifiable risk factors for cognitive decline, particularly in relation to memory and computer-based exercises for cognition. These findings support future research efforts into the development, implementation, feasibility, and acceptability of eHealth interventions to support the health and well-being of individuals with cognitive impairment and their carers. As part of this process, it will be important to develop strategies to promote the use of eHealth technologies, including social media websites and apps among older adults with lower levels of education.

## Appendix

Multimedia Appendix 1 The Healthy Brain Ageing E-Health Questionnaire.

## Abbreviations

eHealth: electronic health
HBA: Healthy Brain Ageing
MCI: mild cognitive impairment
MMSE: Mini-Mental State Examination
SE: standard error
SCI: subjective cognitive impairment
SPSS: Statistical Package for the Social Sciences
