# Supplementary material for: Examining Internet and eHealth Practices and Preferences: Survey Study of Australian Older Adults With Subjective Memory Complaints, Mild Cognitive Impairment, or Dementia
Source: J Med Internet Res. 2017 Oct 25;19(10):e358. doi: 10.2196/jmir.7981 (PMC6257301; doi:10.2196/jmir.7981)
Supplement: Multimedia Appendix 1 [file jmir_v19i10e358_app1.pdf]

HBA e-health questionnaire

We are interested to know about your use of technologies to manage your health and mental health:

Access

1. Do you use a computer?
   - ☐ Yes -If yes, for how many years have you used a computer? ______(specify number of years)
     -If yes, over the last month, on average, how many times did you use the computer per week?
       - ☐ Less than once per week
       - ☐ 1 -2 times per week
       - ☐ 3 – 4 times per week
       - ☐ 5 – 6 times per week
       - ☐ 7 – 8 times per week
       - ☐ 9 – 10 times per week
       - ☐ More than 10 times per week
   - ☐ No, and I'm not interested in using one
   - ☐ No, but I would like to learn
   - ☐ No, I don't have access to one

2. Do you have a computer (desktop or laptop) at home?
   - ☐ Yes
   - ☐ Yes, I have a computer as well as a tablet (e.g. iPad)
   - ☐ No. If no, do you have access to a computer at another location (e.g. family member's home, library)? YES / NO (please circle)

3. Do you have a mobile phone?
   - ☐ Yes
   - ☐ Yes, I have a smartphone (i.e. one that can be used to access the Internet (e.g. Samsung, Blackberry, iPhone)
   - ☐ No, go to question 5

4. Do you use text messaging (SMS)?
   - ☐ Yes
   - ☐ No
   - ☐ No, but I would like to learn

5. Do you have access to the Internet at home (answer ONE only)?
   - ☐ No, and I don't want it (go to question 8)
   - ☐ No, but I wish that I did (go to question 8)
   - ☐ Yes, but I do not use it
   - ☐ Yes, I use the internet independently and have no difficulties
   - ☐ Yes, but computer hardware difficulties prevent me from using it regularly
   - ☐ Yes, but lack of skills/confidence prevent me from using it regularly
   - ☐ Yes, but the internet connection is not good
   - ☐ Yes, but I need someone to assist me

6. If you answered "Yes" to Question 5, how do you connect your computer to the internet at home?
   - ☐ Dial-up connection through your home phone line
   - ☐ Broadband / DSL
   - ☐ Cable (through your tv)
   - ☐ Fibre
   - ☐ Dongle
   - ☐ Mobile hotspot
   - ☐ Don't know

HBA e-health questionnaire

We are interested to know about your use of technologies to manage your health and mental health:

7. Are you satisfied with the speed of your internet connection (e.g. webpages do not take longer than 2 minutes to load, webpages do not crash frequently, etc.)?
   - ☐ Yes
   - ☐ No

8. What is your preferred method of accessing the internet?
   - ☐ Computer (desktop or laptop)
   - ☐ Tablet
   - ☐ Mobile phone
   - ☐ I don't access the internet

9. Do you use email?
   - ☐ Yes
   - ☐ No

10. Do you use any of the following websites (tick ALL that apply)?
    - ☐ Facebook
    - ☐ Twitter
    - ☐ Instagram
    - ☐ Pinterest
    - ☐ LinkedIn
    - ☐ Google
    - ☐ Yahoo
    - ☐ MSN
    - ☐ Other please specify.______________

11. If you answered 'Yes' to questions 9 or 10, please rank from 1 (most frequent) to 5 (least frequent) the following items in order of how frequently you use the internet for these purposes:
    _____ Email
    _____ Social media (e.g., Facebook, Twitter, Instagram, Pinterest, LinkedIn, etc.)
    _____ Searching for information about activities (e.g., events in your area, travel, etc.)
    _____ Searching for information about your health (e.g., medical conditions, medications, practitioners, etc.)
    _____ Reading the news or keeping up with current events

12. What are your favourite websites? Which websites do you visit most frequently?

What are your top 5 websites?	Why (e.g., informative, entertaining, social connectedness, etc.)?

HBA e-health questionnaire

We are interested to know about your use of technologies to manage your health and mental health:

Health management

13. Do you book medical appointments online?
    - ☐ Yes
    - ☐ No. If no, would you do so if this was available? YES / NO (please circle)

If you **do not** have access to a computer, please answer questions *as if you were provided with access to the internet.*

14. Do you currently visit websites that provide health information?
    - ☐ Never - If never, would you do so, if these provided a comprehensive individualised program for you?
      YES / NO (Please Circle) (Now go to question 16)
    - ☐ On occasion (once per month)
    - ☐ Regularly (at least once per week)

15. If you answered 'On occasion' or 'Regularly' to question 14, how confident are you that the health information you access on the internet is reliable? (Please Circle) (Now go to question 17)

Not at all confident		Reasonably Confident		Very Confident
1	2	3	4	5

16. If you answered 'Never' to question 14, what are the barriers that prevent you from using health-related websites?
    - ☐ Lack of confidence in the information
    - ☐ It is too difficult to find information online (e.g., too confusing, too complex, etc.)
    - ☐ The health websites are visually unappealing (e.g., bright colours, too much text, animation, etc.)
    - ☐ I am not interested in finding health information online

17. Would you engage in a *Healthy Brain Ageing* website that provided a program tailored to your needs, such as interventions to help you improve your ability to manage your health or improve cognition?
    - ☐ YES
    - ☐ NO
    - ☐ Maybe

18. Would you use a *Healthy Brain Ageing* internet website to measure changes in your cognition (memory and thinking skills) over time?
    - ☐ Yes
    - ☐ No
    - ☐ Maybe

19. Would you use the internet to measure changes in your mood?
    - ☐ Yes
    - ☐ No
    - ☐ Maybe

20. Would you use the internet to receive programs or interventions for the following (tick ALL that apply):
    - ☐ Mood
    - ☐ Sleep
    - ☐ Exercise
    - ☐ Nutrition
    - ☐ Social outlets, e.g. outings, public talks and seminars, groups
    - ☐ Management of vascular risk factors (e.g high blood pressure, cholesterol, etc.)
    - ☐ Practical strategies for my memory
    - ☐ Online computer exercises for my cognition
